# Challenges of CRISPR-Based Gene Editing in Primary T Cells

**DOI:** 10.3390/ijms23031689

**Published:** 2022-02-01

**Authors:** Alaleh Rezalotfi, Lea Fritz, Reinhold Förster, Berislav Bošnjak

**Affiliations:** 1 Institute of Immunology, Hannover Medical School, 30625 Hannover, Germany; Rezalotfi.Alaleh@mh-hannover.de (A.R.); Fritz.Lea2@mh-hannover.de (L.F.); Foerster.Reinhold@mh-hannover.de (R.F.); 2 Cluster of Excellence RESIST (EXC 2155), Hannover Medical School, 30625 Hannover, Germany; 3 German Centre for Infection Research (DZIF), Partner Site Hannover, 30625 Hannover, Germany

**Keywords:** adoptive T-cell therapy, CAR T cells, CRISPR/Cas9, gene modifications, T cells

## Abstract

Adaptive T-cell immunotherapy holds great promise for the successful treatment of leukemia, as well as other types of cancers. More recently, it was also shown to be an effective treatment option for chronic virus infections in immunosuppressed patients. Autologous or allogeneic T cells used for immunotherapy are usually genetically modified to express novel T-cell or chimeric antigen receptors. The production of such cells was significantly simplified with the CRISPR/Cas system, allowing for the deletion or insertion of novel genes at specific locations within the genome. In this review, we describe recent methodological breakthroughs that were important for the conduction of these genetic modifications, summarize crucial points to be considered when conducting such experiments, and highlight the potential pitfalls of these approaches.

## 1. Introduction

Immune protection relies on a functional adaptive immune system. Its important parts are T cells characterized by the expression of a unique clone-specific cell-surface protein complex, the T-cell receptor (TCR). T-cell-mediated immunity is induced by TCR recognition of peptide antigens derived from intracellularly processed proteins and presented in the context of major histocompatibility complex molecules on the surface of antigen-presenting cells. Rare activated antigen-specific T cells initially differentiate and proliferate in the lymph nodes before traveling to the site of inflammation to exert their effector functions. There, cytotoxic CD8^+^ T cells are crucial for the elimination of intracellular pathogens, while the production of highly specific antibodies from B cells and coordination of innate immunity is impossible without CD4^+^ T cells.

Not surprisingly, individuals with a compromised T-cell immunity, due to either primary immune defects or immunosuppression, are highly susceptible to a variety of microorganisms, which often induce severe disease. For example, human cytomegalovirus (HCMV) infection in healthy individuals is usually asymptomatic. However, in immunosuppressed organ-transplant patients, HCMV induces a severe disease, reducing transplant survival and leading to increased mortality [1,2,3]. Besides immunosuppressive treatments, certain viruses strongly impair the T-cell functions, such as the human immunodeficiency virus (HIV), which leads to the depletion of CD4^+^ T cells. Immunodeficiency can also be a consequence of malignant tumors. Tumor cells, if they evade initial immune surveillance, create a microenvironment that obstructs and hinders antitumor immune responses [4]. To suppress T-cell immunity, tumor cells exploit a self-regulatory mechanism for T-cell over-activation via the checkpoint receptors. Expression of checkpoint receptors on T cells, including cytotoxic T-lymphocyte antigen-4 (CTLA-4) and programmed cell death protein 1 (PD-1), is central to regulating inflammation and induction of peripheral tolerance [5,6]. Upregulation of checkpoint molecule ligands on tumor cells, thus, leads to T-cell exhaustion and anergy. Their blockade recently became the basis for immune-checkpoint therapies [5,6,7].

Another promising approach for treating immunocompromised patients that lack functional T-cell responses has been the adoptive transfer of T cells. Transferred T cells provide protective immunity against harmful complications caused by infectious diseases or malignant tumors (reviewed in References [5,8]). For the treatment of viral infections, adoptively transferred HCMV-specific T cells to human transplant patients reduced viral loads and controlled viremia [9,10,11,12,13]. In tumor patients, initial clinical studies indicated the effectiveness of autologous interleukin (IL)-2-expanded tumor-infiltrating (and thus tumor-antigen specific) lymphocytes for the treatment of solid cancers [14,15], or ex vivo expanded virus-antigen-specific T cells for treatment of Epstein–Barr-virus-induced lymphomas [16] and leukemia [17]. Although those cells proved to be clinically effective at least against certain tumor types [18,19], their widespread use was limited with the number of cells available for adoptive transfer and challenges related to their expansion. These limitations could finally be overcome by the generation of T cells expressing a chimeric antigen receptor (CAR). CARs usually contain a tumor antigen-binding domain, primarily derived from the variable regions of monoclonal antibodies directed against the specific target that is integrated into signaling domains of the T-cell receptor and co-stimulatory molecules important for T-cell activation (reviewed in References [5,20]). CD19-targeting CAR T cells are an FDA-approved treatment for different B-cell malignancies, including B-cell acute lymphoblastic leukemia and non-Hodgkin’s lymphoma [21,22,23,24].

Of note, the first two CAR-T products approved by FDA were produced by lentiviral (Kymriah) or gamma-retroviral (Yescarta) transduction [25], vectors that provide a simple way to package (up to 4.5 kb) double-stranded-DNA templates [26]. However, the production of viral vectors is often time-consuming and associated with significant costs [27,28]. In addition, as the place of the integration into the genome cannot be controlled [29], the use of viral vectors is accompanied by the risk of insertional oncogenesis. Therefore, the patients receiving Kymriah or Yescarta CAR-T cells have to be monitored for more than a decade for the occurrence of undesired events [28,30].

For precise manipulation of specific genomic sites, several genome-editing methods have been developed, including zinc-finger nucleases (ZFNs), transcription activator-like nucleases (TALENs), and the CRISPR/Cas system (CRISPR, clustered regularly interspaced short palindromic repeats; Cas, CRISPR-associated) (reviewed in Reference [31]). ZFNs are recombinant proteins composed of a bacterial restriction enzyme (Fok1) that serves as a nuclease and a DNA-binding zinc finger domain [32,33]. The zinc-finger domains were first identified in the DNA-binding domain of sequence-specific eukaryotic transcription factors [34] and later shown to be modifiable to specifically recognize different DNA sequences. In ZNFs, zinc-finger domains guide Fok1 to induce double-strand breaks (DSBs) at specific sites into the genome, thus inducing genome editing by provoking cellular repair mechanisms. The advantage of ZFNs for genetic engineering is the use of relatively small molecules that can be easily packed into viral vectors. However, the need of designing and constructing a specific nuclease pair for each new gene target represents a significant hurdle for the widespread use of ZNFs. The development, application, and design of ZFNs were reviewed in detail earlier [35,36].

Similar to ZFN, TALENs contain the bacterial cleavage domain Fok1 to induce DSBs. However, in the case of TALENs, the DNA-recognition domain is obtained from a transcription factor produced by plant pathogenic bacteria [37,38,39]. In contrast to ZFNs, TALENs are large size molecules, and their size complicates their delivery to target cells. However, TALENs are easily designed and were shown to work more precisely when compared to ZFNs [40]. Unfortunately, genome editing via TALENs, as well as via ZFNs, is associated with significant off-target effects [41].

**Figure 1:**
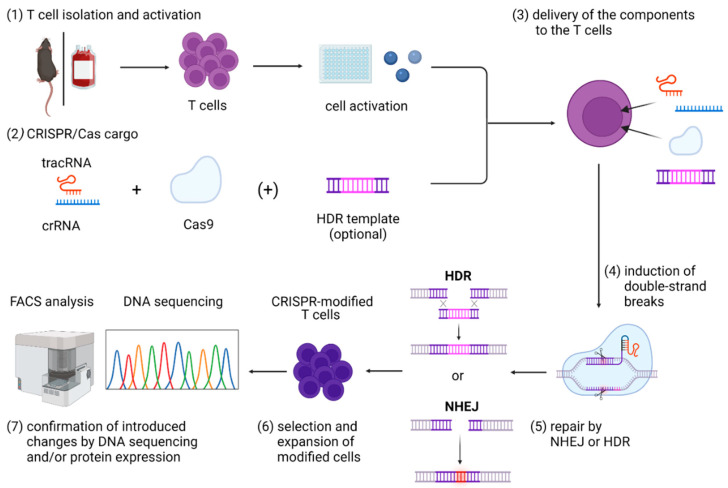
Graphical overview of steps included in the CRISPR/Cas9 gene editing in primary T cells, including (1) T-cell isolation and activation; (2) in vitro assembly of CRISPR/Cas cargo; (3) its delivery to the T cells; (4) induction of double-strand breaks that can be (5) repaired mainly by NHEJ or HDR; and (6) expansion and selection of modified cells and (7) confirmation of introduced changes by DNA sequencing and/or protein expression. Abbreviations: Cas9—CRISPR-associated protein 9; crRNA—CRISPR RNA; HDR—homology-directed repair; NHEJ—non-homologous end joining; tracrRNA—trans-activating CRISPR RNA. Figure created with BioRender.

While both ZNFs [42] and TALENs [43] have successfully been used for editing of T-cell specificity, the CRISPR/Cas system has become the focus of modern gene-editing research during the last decade. Due to its simplicity, the CRISPR/Cas system has established itself as a standard method for genetic modification of almost any cell type, including T cells. This bears enormous potential, both for research and for clinical applications. In the subsequent chapters, we tried to provide a systematic overview of current practices used for targeted genome modification of T cells, using the CRISPR/Cas system (Figure 1). For a more detailed comparison of gene editing via ZFNs, TALENs, and the CRISPR/Cas system, we point the reader to previous reviews [31,44].

## 2. CRISPR/Cas System Origin, Structure, and Molecular Mechanisms of Genome Editing

The CRISPR/Cas system originated from a defense mechanism originally found in bacteria and archaea to counteract infections with viruses known as bacteriophages [45,46,47,48]. The system consists of the Cas protein that has endonuclease activity and the guide RNA (gRNA) that guides the Cas protein to the correct cleavage site in the genome [49]. The gRNA is formed by two distinct RNA molecules, the trans-activating CRISPR RNA (tracrRNA) and the CRISPR RNA (crRNA) [50]. In bacteria and archaea, crRNAs are parts the genomic information of viruses that infect these microorganisms. During bacteriophage infection, the microorganisms have to cut and incorporate the crRNAs into their own genome. The crRNA is expressed, bound to tracrRNA, and loaded onto the Cas protein to guide this nuclease to the complementary sequences in the genome of the attacking virus during re-infection. After the crRNA binds to a complementary sequence, the Cas protein needs to recognize a protospacer-adjacent motif (PAM) in the target sequence to specifically cut and destroy the viral genome, allowing for the protection of the bacteria [46]. Importantly, by providing the chemically synthesized gRNAs, the Cas protein can be programmed to target a specific site in the genome [50]. Therefore, targeted transfer of the CRISPR components allows for the induction of precise DSB and, eventually, gene editing in eukaryotic cells [49,51]. Of all Cas proteins discovered so far [52], the most frequently used for gene modifications in eukaryotic cells is the Cas9, originally found in *Streptococcus pyogenes* [53]. Cas9 was selected because of its multiple properties. In contrast to class 1 CRISPR systems in which the effector molecules are assembled as multi-subunit effector protein complexes, the Cas9, belonging to class 2 CRISPR systems, is a single large effector protein. Even more important is the simplicity and frequent genomic occurrence (on average, once in each 8 bp) of its PAM sequence (5′-NGG-3′), which offers wide genome-editing possibilities [54]. Furthermore, the CRISPR/Cas9 system is not affected by the methylation status of the DNA [55]. Cas9 is now a workhorse for genome modification and is directed to specific locations in the genome by modulation and artificial synthesis of crRNAs [50], allowing for the targeted modification of single nucleotides or entire genomic regions. This review, therefore, focuses on results generated with this nuclease.

Targeted by a crRNA to a specific location within the genome, Cas9 cuts the DNA 3 nucleotides (nt) upstream of the PAM sequence. The resulting Cas9-induced DSB initiates eukaryotic cell-repair mechanisms that prevent cell death due to the loss of genetic material (reviewed in detail in Reference [56]). Two main repair mechanisms exist in eukaryotic cells known as non-homologous end joining (NHEJ) and homology-directed repair (HDR). DSB-repair by Ku-protein-dependent classical NHEJ can be divided into different phases, starting with the recognition of the DSB and assembly of the NHEJ complex, followed by bridging, and stabilization of the DNA ends. After this, the DNA-ends are processed and ligated before the NHEJ complex dissolves [57,58]. However, NHEJ does not use a template for DNA repair, insertions, and/or deletions (indels) of genomic material can occur. Depending on the cell type and its status, 20–50% of repaired DNA molecules will contain indels [59,60,61,62].

If the Ku protein is not expressed, the resection machinery exposes extensive single-strand DNA (ss-DNA) on both sides of DSBs, allowing the cells to use microhomology-mediated end joining (MMEJ) or HDR for DNA repair. MMEJ is an alternative NHEJ mechanism that is initiated if the DNA on both sites of the DSB contains microhomologous sequences. Annealing of those sequences leads to the removal of heterologous overhangs, fill-in synthesis, and ligation (reviewed in Reference [63]). In contrast to MMEJ, HDR uses a homologous DNA template to repair a DSB. One of the first events during HDR is the activation and recruitment of phosphorylases, such as ataxia-telangiectasia-mutated protein (ATM) and Rad3-related kinase (ATR) to the site of damage [64]. These enzymes phosphorylate several proteins, including the DNA-stabilizing histone H2AX on serine 139 [65,66,67], which seems to play an important role in the recruitment of additional repair and signaling molecules to the DSB [68]. As reviewed in Marini et al., the DNA ends at the DSB are then processed through different nucleases and helicases to create a 3′ single strand (ss) DNA that is bound by the replication protein A (RPA) for stabilization [69]. RPA is next replaced by DNA repair protein RAD51 homolog 1 (RAD51) that mediates invasion of ss-DNA filament on the homologous DNA template, usually the complementary strand of the sister chromatid DNA duplex, which is then is used to synthesize the missing genomic parts [70,71]. This mechanism of HDR is now exploited for repairing CRISPR/Cas-induced DSBs. Targeted delivery of synthetic DNA provides an artificial HDR template that can be used to specifically insert genetic information by the cells’ own repair mechanisms. As explained in detail in Section 5, the resulting gene-editing changes can encompass single-nucleotide substitutions, as well as insertions of whole genes.

NHEJ ligates the blunt-end DNA fragments fast and independent of the cell cycle and is, therefore, the favored way for DSB-repair in mammalian cells [72,73]. In contrast, MMEJ and HDR are much slower and restricted to the S and G2 phases of the cell cycle [74,75,76,77,78]. Therefore, genome editing in the presence of a DNA template usually leads to a combination of HDR edits and indels resulting from an NHEJ. The choice between NHEJ and HDR is influenced by different factors at various stages of the repair pathways, as reviewed by Yang and colleagues [76]. For example, cyclin-dependent kinases (CDKs) play a major role in choosing a defined repair pathway. CDK-mediated phosphorylation of different substrates, such as the carboxy-terminal binding protein-interacting protein (CtIP), in the S phase of the cell cycle promotes HDR. In contrast, reduced CDK-activity in the G1-phase promotes NHEJ [77,78]. In addition, repair pathway choice is influenced by DNA-end resection. For HDR initiation, the Ku-protein molecule complex needs to dissociate, and this is achieved by different ubiquitination events, making it a crucial factor for repair pathway choice [79]. Besides Ku proteins, other molecules involved in DNA-end resection that can lead to the selection of a certain repair route are 53BP1 and BRCA1 (reviewed in Reference [80]). Whereas 53BP1 prevents resection of DNA ends and thereby HDR, a BRCA1-dependent process removes 53BP1, leading to the promotion of the HDR-pathway. Therefore, the NHEJ and HDR compete, with molecules of one pathway actively suppressing the other.

## 3. Practical Aspects of CRISPR/Cas9 Gene Editing on T Cells

For successful application of CRISPR/Cas-mediated genome editing by using either the NHEJ or HDR pathway, several practical aspects appear to be critical.

### 3.1. Culturing of T Cells for Genome Editing

Unless stimulated, isolated T cells survive in vitro only for a few days. While human T cells can survive this period without any cytokines added to the medium, mouse T cells require IL-7, which upregulates the expression of the anti-apoptotic protein Bcl-2 and maintains their viability [81]. During this time, unstimulated T cells can be targeted with CRISPR/Cas to some extent to induce gene knockouts [82,83,84].

**Figure 2:**
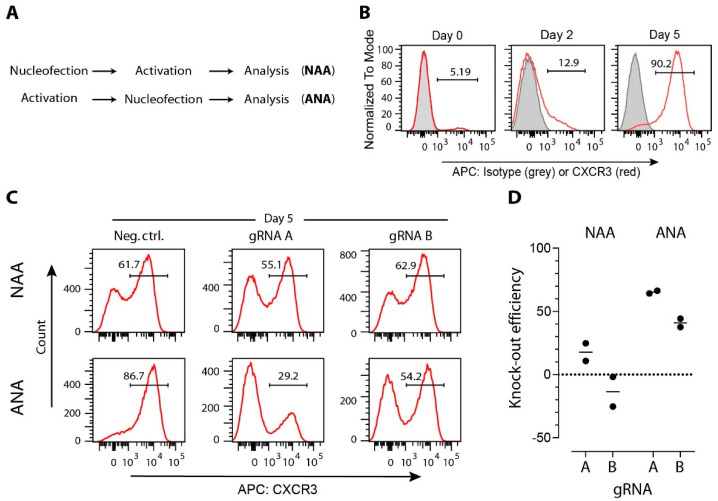
Efficacy of CRISPR/Cas9-induced modifications in T cells is affected by gene expression level. (**A**) CD4+ T cells and MACS-sorted from mice spleen and lymph nodes, were either immediately nucleofected before activation (protocol NAA) or were first activated and then nucleofected (protocol ANA). T-cell activation on anti-CD3/-CD28 coated wells, nucleofection with one two anti-*Cxcr3* crRNAs (A: 5′-TGACTCCCCGCCCTGCCCAC-3′; B: 5′-GCTGTTCTGCTGGTCTCCAG-3′) or with a negative control crRNA (Neg Ctrl: 5′-CGTTAATCGCGTATAATACG-3′) coupled with tracrRNA and Cas9 (all components from Integrated DNA Technologies Inc.) into CRISPR/Cas9 ribonucleoproteins (RNPs), and expansion in medium supplemented with 100 U/mL of interleukin-2 was performed according to our previously published protocol [99]. (**B**) Representative histogram plots showing CXCR3 expression on CD3^+^CD4^+^ T cells immediately after isolation (day 0), after anti-CD3/-CD28 activation (day 2), or after additional 3 days of expansion (day 5). (**C**) Representative histogram plots showing CXCR3 expression on CD3^+^CD4^+^ T cells nucleofected with indicated Cas9 RNPs. (**D**) Gene-editing efficacy expressed as a relative decrease of CD3^+^CD4^+^CXCR3+ cells after nucleofection anti-*Cxcr3* crRNAs compared to negative control group. Data are from two independent experiments (dots), and group means are shown as lines.

Unstimulated T cells are in a resting state, and many genes are not actively transcribed. It has been shown that Cas9 binding is affected by DNA packaging. Actively transcribed genes can be more efficiently edited than non-actively transcribed genes where the DNA is packaged in heterochromatin (reviewed in Reference [85]). Similarly, gRNAs targeting divergent regions within the same gene may also result in different efficiencies due to local variation in chromatin structure [86]. It is not surprising, therefore, that the activation of T cells before gene manipulation can significantly increase the efficiency of CRISPR/Cas9 gene editing with the same set of gRNAs (Figure 2).

Gene editing via CRISPR/Cas-mediated HDR is considered not to be feasible in resting cells, which are in the G1 and not the S/G2 phase of the cell cycle [77,78]. However, it was recently reported that naive T cells can be transfected with HDR templates and gRNA/Cas9 ribonucleotide particles (RNPs) and that following adoptive transfer into recipient mice CRISPR/Cas9-based HDR could be achieved [83]. However, the recipient mice were immunized immediately upon cell transfer, leading to T-cell activation and proliferation, so it is still questionable whether HDR integration actually occurred in the naive or in the activated T cells. It is, therefore, still generally accepted that induction of T-cell proliferation is necessary for HDR-mediated gene editing [25,87].

Most gene-editing experiments were performed on in vitro activated T cells, and a large number of divergent experimental protocols used to activate T cells in vitro have been reported in the literature. The most frequently used protocols involve non-specific T-cell activation, using anti-CD3 (and anti-CD28) antibodies coated on the surface of culture vessels or provided pre-bound to beads [88]. Performed under optimal conditions, these protocols strongly activate T cells and induce their proliferation, albeit proliferation induced with the beads seems to be slightly stronger [89,90]. Results from different studies can hardly be compared, as they are often conducted in different media supplemented with divergent cytokine cocktails, including IL-2, -7, and/or -15 [88,91]. Of note, the efficiency of NHEJ-mediated gene editing has been successfully performed in T cells activated in antibody-coated culture vessels [82,92] or beads [93]. In contrast, the efficiency of HDR-mediated gene editing seems to be superior in T cells activated with protocols applying beads [94]; however, a recent preprint has indicated the opposite [95]. As a starting point, we refer the reader to few most recent protocols [96,97,98].

### 3.2. gRNA Selection

Another point that needs to be taken into account when performing CRISPR/Cas experiments is the efficacy and specificity of the respective gRNA. To evaluate on-target efficacy, numerous computational tools for gRNA scoring have been developed and recently reviewed [100,101]. Due to the differences in datasets and models used for the tool creation, it is suggested that the gRNAs should be selected by using several divergent tools and then further evaluated in preliminary experiments [100].

Besides efficacy, the off-target activity of the CRISPR/Cas9 system is still a great concern, especially regarding its clinical applications. Therefore, the detection of off-target effects is essential for the estimation of the precision of gene-editing, as well as for improvement. Off-target effects can be detected via different methods, including the T7 endonuclease I assay (can detect off-target mutation frequencies <1%) [102], deep sequencing [103], and genome-wide unbiased identification of DSBs enabled by sequencing (GUIDE-seq; detects mutation frequencies of 0.12%) [104]. For further reading about the mentioned methods to detect off-target effects and strategies to reduce off-target effects, we point the reader to previously published reviews [105,106].

### 3.3. CRISPR/Cas Cargo

Besides selecting an in vitro protocol for T-cell activation, it is also important to consider in which form the CRISPR/Cas9 components get delivered into the target cells. In the case of gene-knockout experiments, gRNA and Cas9 can be delivered as plasmids, as mRNA encoding for Cas9 with separate delivery of gRNAs, or in the form of ribonucleoproteins (RNPs). Initially, Cas9 and the gRNA were incorporated into plasmids for cell delivery. However, delivery of high amounts of plasmid DNA can also have toxic effects [107], mostly due to the recognition of pathogen-associated molecular patterns within the plasmid DNA [108,109]. Furthermore, plasmid delivery poses the problem of random DNA integration into the cellular genome [110]. Alternatively, Cas9 can be delivered in form of an mRNA [111,112]. Furthermore, to prevent cytotoxicity, the mRNA can be synthetically modified by the addition of a 5′-methylated cap and a 3′-poly-A tail, making the mRNA more similar to endogenous mRNA [113]. This approach was also successfully used in human T cells [92], albeit it usually involves gRNA delivery in a separate transfection step [111,112].

Several current reports used preformed RNPs that allow for a faster onset of action and reduced off-target DNA cleavage without risk of integration into the genome [82,94,110,114,115]. For RNP construction, the specific crRNA and the tracrRNA are either in vitro annealed to form the gRNA or already synthesized as a single gRNA molecule, which is then loaded onto the Cas9 protein to form the RNP complex.

Besides Cas9 and gRNA, an HDR template also has to be provided to the cells to incorporate genetic material by using the CRISPR/Cas system. HDR templates can be provided as a single-stranded DNA (ss-DNA), long linear or circular (plasmid) double-stranded DNA (ds-DNA), or AAV-incorporated DNA molecule [116]. All three template types have been used successfully to make changes into the T-cell genome, as is discussed in detail in subsequent sections.

### 3.4. Strategies for Delivery of CRISPR/Cas Cargo to T Cells

Existing delivery approaches can broadly be divided into viral and non-viral strategies. Viral strategies use transduction of cells with viral vectors to deliver DNA, encoding the sequence of the Cas protein, as well as of the gRNA. The most frequently used vectors are derived from ADVs, AAVs, or lentiviruses. While lentiviral vectors for CRISPR/Cas components carry the oncogenic potential, the use of adenovirus (ADV) and adeno-associated virus (AAV) vectors is accompanied by concerns regarding specific ADV immunity in humans, which could compromise the engraftment of those cells in clinical settings (reviewed in Reference [117]). Therefore, direct delivery of non-virally packed ds-DNA is preferred for clinical applications [117], increasing interest in developing suitable non-viral delivery strategies. For further insight on these different strategies of viral delivery, we refer the reader to a recent review by Xu et al. [26].

The literature reports an increasing arsenal of non-viral delivery strategies that successfully supply components of the CRISPR/Cas system into the cells (reviewed in References [25,118,119]). Several techniques suitable for transfection of other cell types did not prove to be applicable for T cells. For example, microinjection, which uses microscopic needles for the injection of molecules into the nucleus of individual cells, is extremely efficient but technically challenging and laborious, and, thus, it is restricted to the generation of transgenic animals [120,121]. Further, T cells are resistant to lipofection, a method based on the delivery of liposome- or lipid-nanoparticle-encapsulated contents to the target cells [122]. Gold nanoparticle-mediated techniques, based on the cellular uptake of biological material enwrapped around a gold core, successfully delivered CRISPR/Cas9 components to T cells, albeit with low efficacy [123].

In contrast, electroporation and nucleofection allow efficient delivery of different types of nucleic acids to T cells. Both techniques use electrical current to induce pores in the cell membrane that allow cargo entry into the cells by diffusion and/or movement along the electrical field [124]. In contrast to electroporation, nucleofection uses a combination of cell-type-specific electrical pulses and reagents to deliver molecules directly into the nucleus [125]. An initial study showed efficient delivery of Cas9 mRNA and gRNA to primary T cells via electroporation [126]. However, in 2018, Akiko Seki and Sascha Rutz published a seminal paper in which they described an optimized protocol for CRISPR/Cas9 RNP delivery by nucleofection to primary T cells [82]. Since then, this technique has become most frequently used to deliver the CRISPR/Cas cargo to T cells, due to its simplicity and effectiveness [94,114,127,128,129]. For this, Cas9 RNPs are prepared with either a single gRNA or a combination of multiple gRNAs targeting the same gene. Although RNP with a single gRNA can efficiently disrupt the target gene [110,114], more recent studies have shown that using multiple gRNA directed against the same target strongly increases the knockout efficiency, resulting in almost complete loss of the molecule expression [82,99]. These findings are also consistent with our observations [99,130]. Recently, it has been suggested that the efficacy of this method can further be improved by encapsulating Cas9 RNPs into poly-L-glutamic acid (PGA) nanoparticles [115].

Nucleofection (and electroporation) leads to a considerable loss of cell viability and also affects T-cell activation [131,132]. Therefore, there is still room for developing alternative delivery methods that are able to match the high efficiency of nucleofection but without its detrimental side effects. One alternative could be cell squeezing, which relies on a temporary mechanical membrane disruption to deliver molecules to the cells by diffusion [133]. Another delivery strategy induces transduction by osmocytosis and propanebetaine (iTOP), which is based on a cellular uptake mechanism called macropinocytosis [134]. This method was recently shown to efficiently deliver RNPs to human cells, including primary T cells [135].

## 4. Gene Modifications in T Cells by NHEJ-Mediated Repair of CRISPR/Cas9-Induced DSBs

In the absence of an HDR template, the cell relies almost exclusively on NHEJ to repair DSBs introduced by CRISPR/Cas9. As mentioned before, this can lead to indels in the coding region of a gene, resulting in frameshift mutations and disruption of protein function. This is why the CRISPR/Cas9 system is widely used in modern gene-editing research. It facilitates the creation of knockout cell lines or animals for investigation of disease mechanisms, as well as providing the basis for the development of therapeutic strategies.

Regarding T cells, CRISPR/Cas-induced knockouts have manifold applications in basic research, enabling elegant ways to study the role of individual molecules or complete signaling pathways. For example, we analyzed the mechanism of T-cell homing to the lymph nodes after intra lymphatic injection, which allows us to study the entry of T cells into lymph nodes that arrive via afferent lymph [99]. Using a lentiviral CRISPR/Cas approach, we simultaneously knocked out four integrin genes (*Itgb1*, *Itgb2*, *Itgb7*, and *Itgav*). Since integrins form dimers on the cell surface, the simultaneous knockout of these four integrin genes prevents the expression of any integrin on immune cells. By using complete integrin-knockout CD4^+^ T cells or CD4^+^ T cells that lack the integrin-associated adaptor molecule Talin 1, we could establish that integrins contribute to T cells entry into the lymph node parenchyma and translocation to the T cell zone of the lymph node [99].

The CRISPR/Cas9 system can also be used as a large-scale loss-of-function screening method (reviewed in Reference [136]). For this, several libraries of gRNAs designed to target every gene in the mouse or human genome are commercially available. These gRNAs are then virally transduced into the cells so that each cell receives only a single virus, carrying one gRNA. After selection and expansion, the transduced cells are then used in a positive or negative screen, allowing for the identification of a phenotype of interest. Next-generation sequencing of input and output cell populations is used to identify the gRNAs that targeted genes causing the desired phenotype. Several such CRISPR screens have been performed on T cells, including one used to analyze genes involved in T-cell activation [137]. Transduction of Cas9-expressing Jurkat cells with a lentiviral sgRNA library confirmed known regulators of T-cell activation (such as CD3, Lck, and Zap70), as well as unraveled FAM49B as another regulator of T-cell activity [137]. CRISPR screens were also used to identify gene-regulatory programs that promote or disrupt Foxp3 expression in regulatory T cells [138]. More recently, a CRISPR screen on CD8^+^ T cells indicated that their effector functions are negatively regulated by transcription factor Fli [139]. These examples show that CRISPR screens hold enormous potential for the discovery of genes contributing to various aspects of T-cell function.

In addition to its impact on basic research, the NHEJ-mediated gene knockout by CRISPR/Cas system also opens up new possibilities for clinical applications, including cancer therapy. Since certain tumor cells express ligands for exactly these immune checkpoint molecules [140], the effective antitumor response by CAR T cells is inhibited [141,142]. The CRISPR/Cas system can be used to knock out immune checkpoint molecules to overcome checkpoint molecule mediated inhibition of tumor-infiltrating lymphocytes and CAR T cells. This approach showed promising results in different preclinical studies in which CRISPR/Cas9-generated PD-1 knockout T cells showed enhanced antitumor activity [143,144,145]. The safety of transferring genetically modified T cells to patients was recently tested in clinical trials on metastatic non-small lung cancer patients (clinical trial information: NCT02793856) [146]. In addition, a pilot study in three patients with advanced refractory cancer was performed [147]. In that study, adoptive transfer of TCR and PD-1 knockout CAR T cells targeting the NY-ESO-1 tumor antigen resulted in the homing of these cells to the tumor site and tumor size reduction. Furthermore, monitoring revealed that the cells were persisting for at least 9 months, without inducing clinical toxicity [147]. If the results of other currently ongoing clinical trials (Table 1) confirm these promising preliminary results, CRISPR/Cas9 modified PD-1 KO T cells could become an intriguing treatment option for antiviral therapy, using virus-specific T cells. Initial studies in mice, for example, showed that lack of PD-1 expression on effector T cells resulted in faster control of adenovirus infection [148].

Knocking out genes via the CRISPR/Cas system can also be applied to render transferred T cells insensitive to immunosuppressive therapy. Immunosuppressive treatment after organ transplantation is essential for the prevention of graft rejection or destruction of the transplanted organ by the recipient’s T cells. One of the immunosuppressive drugs, tacrolimus, targets the T-cell adaptor protein FK506-binding protein 12 (FKBP12) and prevents cytokine release and T-cell activation. However, immunosuppressive treatment does not only specifically target the cells that are responsible for transplant rejection but leads to general immunosuppression. Hence, immunosuppressed patients are susceptible to a variety of pathogens, including HCMV. To overcome the problem that virus-specific T cells also become inhibited by tacrolimus upon adoptive transfer, Amini et al. created tacrolimus-resistant HCMV-specific T cells by knocking out *FKBP12* with the CRISPR/Cas system [149]. Importantly, the *FKBP12*^−/−^ HCMV-specific T cells showed effector functions comparable to unmodified virus-specific T cells upon in vitro HCMV-peptide restimulation [149]. Therefore, the production and transfer of tacrolimus-resistant CMV-specific T cells could be a promising approach for HCMV therapy in immunosuppressed transplant patients. Likewise, Jung et al. used CRISPR/Cas9 to knock out diacylglycerol kinase (*DGK*) to increase T-cell-receptor-mediated signaling in CAR T cells [150]. *DGK*^−/−^ CAR T cells were less sensitive to transforming growth factor beta (TGFβ) and other immunosuppressive factors, had enhanced effector functions, and were able to clear tumors in a xenograft mouse model [150]. In another study, the gene for adenosine A2A receptor on CAR T cells was targeted to improve antitumor efficiency [151]. The highly increased concentration of extracellular adenosine in tumors impairs the antitumor activity of T cells upon binding to the adenosine A2A receptor. Knocking out the receptor gene in activated human T cells resulted in enhanced antitumor function of CAR T cells, leading to reduced tumor growth and prolonged survival of tumor-bearing mice [151].

The use of autologous T cells is often restricted by issues related to manufacturing time, scalability, and expenses caused by cell expansion. Therefore, the use of allogeneic T cells from healthy donors for adoptive T-cell therapy would offer several benefits. However, adoptive transfer of allogeneic cells causes graft-versus-host disease (GvHD), due to the recognition of host antigens by transferred T cells. Recently, Kamali et al. successfully used the CRISPR/Cas system to knock out the endogenous T-cell receptor alpha chain constant (*TRAC*) gene on T cells, thus abrogating the expression of the endogenous T-cell receptor (TCR) on the cell surface [152]. In addition, the authors also disrupted the *CD52* gene to render the transferred cells insensitive to anti-CD52-antibody depletion, a procedure frequently used to deplete hematopoietic leukemic cells. However, FACS analysis revealed that only 7–8% of cells completely lost the TCR and the CD52 molecule [152]. Still, these studies indicate the enormous potential of CRISPR/Cas9 for the creation of T cells carrying divergent gene modifications. Their potential has outgrown pure research applications, and several clinical studies are starting (Table 1). Of note, these protocols require rigorous testing of the modified cells to assure that they do not carry any undesired off-target mutations.

**Figure 3:**
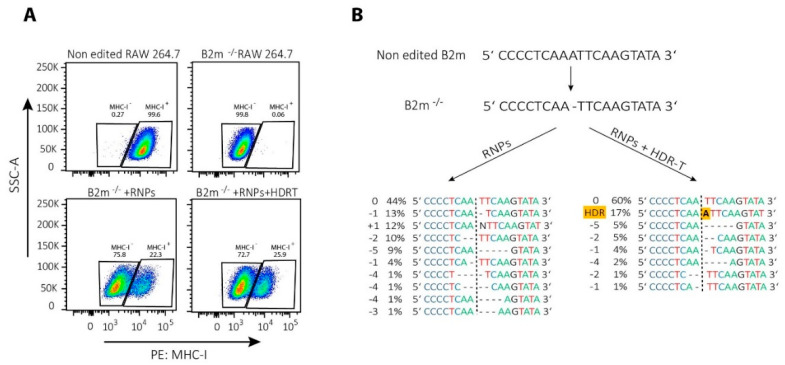
CRISPR/Cas9-mediated correction of frameshift mutations via NHEJ or HDR pathway. To repair a single base-pair deletion in beta-2-microglobulin gene (*B2m*) (NC_000068.8:121981364), cells were nucleofected with CRISPR/Cas9 ribonucleoparticles (RNPs) containing 70 pmol of Cas9 complexed with 210 pmol of crRNA (5′-GCGTGAGTATACTTGAATTG-3′):tracrRNA complexes together with 70 pmol of electroporation enhancer or with the same RNPs in the presence of 100 pmol of single-stranded DNA HDR template (5′-GTTTTCATCTGTCTTCCCCTGTGGCCCTCAGAAACCCCTCAAATTCAAGTATACTCACGCCACCCACCGGAGAATGGGAAGCC-3′; all components from Integrated DNA Technologies Inc.). Nucleofection with prepared RNPs with or without HDR template in a total volume of 10 µL was performed by using SF cell line 4D-NucleofectorTM X Kit L and a 4D nucleofector X and Core units (all Lonza), using program for RAW 264.7 cell (pulse code: DS136). After nucleofection, cells were expanded for 2 days before they were used for (**A**) FACS analysis of major histocompatibility complex class I (MHC-I) re-expression of the cell surface; or (**B**) DNA extraction to amplify by PCR the region of interest (Forward primer 5′-GACACTGCTAAAAGCCAGGT-3′, reverse primer 5′-CAGATGGAGCGTCCAGAAAGT-3′; 98 °C 30″, 35 cycles (98 °C 5″, 55 °C 10″, 72 °C 40″), 72 °C 2′, using high fidelity DNA polymerase), sequencing it by Sanger sequencing and analyzing the results by using ICE (Synthego) to determine the type of genetic changes induced by NHEJ or HDR.

It is important to note that NHEJ-mediated repair of CRISPR/Cas9 induced DSBs is not stochastic but, rather, a predictable process [153,154]. Therefore, even NHEJ-mediated repair can be successfully used for template-free correction of pathogenic frameshift or micro-duplication mutations [153]. In T cells, Roth et al. corrected single mutations in patients with IL-2 receptor (*IL-2R*) coding gene immunodeficiency by NHEJ-mediated repair alone, possibly due to the introduction of small indels that restored an appropriate reading frame [94]. Similarly, we noticed that a single nucleotide deletion in the beta-2-microglobulin (*B2m*) gene can be repaired by HDR template-dependent and -independent mechanisms, as revealed by re-expression of major histocompatibility complex class I (MHC-I) on the cell surface (Figure 3A). Results of the DNA sequencing showed that the protein expression was restored mainly due to frameshift mutations, while a provided template has been integrated only into the minority of cells (Figure 3B).

## 5. Gene Knock-in Strategies in T Cells, Using HDR-Mediated Repair of CRISPR/Cas9-Induced DSBs

For precise gene editing, the HDR pathway can be exploited by providing an HDR template together with the gRNA and Cas9, offering an enormous potential in the field of gene therapy (reviewed in References [44,155]). For example, malignant or disease-causing mutations could be excised from the genome and replaced with non-mutated DNA sequences by providing appropriately targeted gRNA, and Cas9 together with a suitable HDR template. Initial studies showed the CRISPR/Cas system can successfully be used to correct genetic mutations from patient-derived induced pluripotent stem cells (iPSCs). For example, Firth et al. used the CRISPR/Cas system for restoring the function of the cystic fibrosis transmembrane regulator (CFTR) in iPSC from a cystic fibrosis patient [156]. Importantly, genetically edited cells were still able to differentiate into mature airway epithelial cells that had normal CFTR function [156]. In another study, CRISPR/Cas9 mediated HDR was used to correct the β-41/42 (TCTT) deletion mutation in β-Thalassemia patient-derived iPSCs that could be differentiated into erythroblasts with normal *HBB* (β-globin) gene expression [157]. Furthermore, Park and colleagues used the CRISPR/Cas9 system to repair the disease-causing chromosomal inversion in iPSCs from hemophilia A patients [158]. Endothelial cells differentiated from those iPSCs expressed the *F8* gene and were also fully functional in a mouse model of hemophilia [158]. Together, these studies indicate the enormous potential of the CRISPR/Cas system for genomic correction of patient-specific iPSCs for the treatment of monogenic disorders. For further information about the applications of CRISPR/Cas9 genome editing in human iPSCs, especially for the discovery of therapeutic approaches and gene therapy, we refer to a recent review by Masi et al. [107].

HDR pathway can be used in T cells for research purposes, including the insertion of fluorescent protein genes (such as GPF) or bioluminescent tags [159]. More importantly, in the field of cancer therapy, the CRISPR/Cas system in combination with HDR was successfully used to insert the CAR gene into the *TRAC* gene, thus disrupting the expression of the endogenous TCR [126,160]. This approach allows the production of T cells that are directed against tumor-specific antigens to effectively treat a variety of malignant diseases. Thus, HDR-mediated CRISPR/Cas modifications hold the promise to become an important tool for the in vitro generation of T cells with various specificities that can be used “off-the-shelf” for immunotherapies. To achieve a successful HDR-mediated gene editing, several key points should be considered during template design and its co-delivery into the cells.

### 5.1. Selection of Appropriate gRNAs for HDR-Mediated Repair

As for gene knockout experiments, the most important parameter for the initiation of HDR seems to be the efficacy of the gRNA (or crRNA/tracrRNA) used [116]. In addition to the gRNA efficacy, it seems that the targeted DNA sequence also affects which type of genome editing occurs. A recent bioinformatic analysis revealed that sequences repaired with MMEJ more often induce HDR than sequences repaired with classical NHEJ [161].

For effective HDR, it also seems to be crucial to select gRNAs that will guide Cas9 to PAM sites at the proper distance from the desired editing site [86]. The gRNA should be designed such that the DNA cut site is as close as possible to the region of homology [162], because knock-in efficiency drops as this distance increases [87]. It has been suggested that the distance between the PAM sequence and the site of the mutation should not exceed 10 bases [163,164]. A study by M. Liu et al. has shown that DSB should be within 10 bases up and to a maximum of 100 bases of the integration site [87]. However, gRNAs with high editing efficiency will induce high HDR insertion rates, even if their cutting sites are more than 10 bases away from the insertion [116].

### 5.2. HDR Template Type

An HDR template can be in a form of ss-DNA, long linear or circular (plasmid) ds-DNA, or AAVs-incorporated DNA [116]. Of note, it is important to protect the ends of linear donor DNA templates with phosphorothioate modifications to prevent them from degradation within the cells [162]. All in all, the literature indicates that all template forms mentioned can successfully be used for gene editing in T cells. Eyquem et al. delivered Cas9 mRNA and gRNA by co-electroporation, following transduction of AAV vector carrying an HDR template to disrupt the *TRAC* gene and replace it with a CD19 CAR with a knock-in efficiency of ≤40% [126]. On the other hand, Roth et al. co-electroporated RNP complexes with ss- or ds-DNA templates to create T cells with various knock-in modifications with efficacies up to ~40% and ~50%, respectively [94]. However, the use of ds-DNA templates (irrespectively of the mode of delivery) has several limitations. These templates are toxic to the cells and have a relatively narrow working concentration [94,95]. Moreover, ds-DNA templates can also be incorporated by dominant NHEJ process or even at off-target DSB [94]. All of these issues can be avoided by the use of ss-DNA templates. However, the synthesis of long (>500 nt) ss-DNA templates is technically challenging and relatively expensive. The choice of the HDR template design seems to be mostly dependent on the insertion size. In general, ss-DNA molecules seem to be optimal templates for the induction of small changes, such as SNPs or short inserts. On the other hand, the ds-DNA donors are preferred for inserts longer than 100 bp, such as epitope tags or entire genes [162].

If ss-DNA are used as HDR templates, it is also important to consider whether it will be synthetized as the sequence of the genomic DNA strand containing the PAM (non-target strand, as it does not bind gRNA) or as the sequence of genomic DNA strand that binds gRNA (target strand). Of note, while Cas9 globally dissociates from duplex DNA symmetrically, after cleavage, it locally first releases the non-target strand before the target strand [165]. Hence, Richardson et al. tested the co-delivery of Cas9 RNP with ss-DNA templates complementary to the non-target or target DNA strand into HEK293 cells and found that the non-target strand donor exhibited on average 2.6x higher HDR efficiency than the target strand donor [165]. However, a subsequent study using a different set of gRNAs in the same cells found that the target strand (without PAM) served as a better donor than non-targeted strand [162]. Similarly, ss-DNA with the sequence of target strand had superior HDR efficacy than the non-target strand ss-DNA in iPSCs [166]. These seemingly contradictory results were recently resolved when it was demonstrated that the donor-strand preference depends on the genome location and the type of cell used [116,167].

### 5.3. Design of HDR Template “Homology Arms”

Irrespective of the HDR type used, it is crucial to properly design its sequences homologous to the specific sequence flanking the cut site (“homology arms”). If working with human material, special care should be taken to assure that these sequences are free of any polymorphisms that could prevent template incorporation. The length of the homology arms depends on the size of the desired change. Large insertions, such as those coding for GFP or a CAR, require templates with homology arms of a length ≥ 300 bp [94,95]. On the other hand, homology arms with a length of approximately 60 bp efficiently introduce short modifications, for example, nucleotide exchanges to correct mutations [94,114]. Actually, for this type of gene editing ss-DNA and linear ds-DNA with relatively short homology arms (up to 80 bp) proved to be equally good [168] or even more efficient than dsDNA plasmid template with long (>500 bp) homology arms [169].

In the case where ss-DNA templates are used, it is important to consider whether symmetric or asymmetric homology arms are used. The latter commonly have a shorter homology arm on the PAM-distal side and a longer homology arm on the PAM-proximal side of the break. The templates with asymmetric homology arms were reported to have enhanced HDR efficiency compared to their counterparts with symmetric homology arms [162,165]. However, it was recently suggested that the asymmetry of homology arms affects only templates with short (<30 nt) homology arms [116].

Recently the group of from Alexander Marson showed that HDR efficiency can be two- to four-fold improved by adding the short truncated Cas9 target sequences (tCTS) to homology arms of ds-DNA templates. The increased HDR efficacy is a result of Cas9-mediated template shuttling into the nucleus, as RNPs can bind to, but not cleave, tCTS [115]. Similarly, a recent preprint also reported that tCTS-modified ds-DNA HDR templates have an advantage over conventional templates for CAR-T gene knock-in, albeit only at low template concentrations [95].

It is also important to incorporate mutations within the HDR template that prevent the re-cleavage by Cas9 once the template got integrated [116,163,166]. Recently, Schubert et al. showed that, in the absence of such mutations, the efficacy of correct template integration is as low as <2% [116]. The addition of a blocking mutation in the second or third base of the “NGG” PAM sequence increased the efficacy of template integration to 8.0–17.8%. Similarly, mutations within the seed region of crRNA (defined as the PAM-proximal 10–12 bases on the 3′ end of the crRNA) also significantly increased template integration efficacy, albeit at a lower rate than the mutations within the PAM sequence [116].

### 5.4. Inhibition of HDR Template Toxic Effects

The delivery of high amounts of ds-DNA can have toxic effects, mostly due to the recognition of pathogen-associated molecular patterns within the plasmid DNA [108,109]. Interestingly, a preprint reported that treating T cells with DNA-sensor inhibitors before electroporation with an HDR template led to a slight increase in cell viability and CAR insertion rate [95]. However, these results seem to be related to a partial increase in the number of T cells being in the S phase of the cell cycle after treatment with DNA-sensor inhibitors, rather than with direct anti-toxic effects [95].

### 5.5. Promoting HDR over NHEJ

The efficacy of HDR can be increased by tipping the choice for DSB repair away from NHEJ (reviewed in Reference [76]). This can be achieved by suppression of NHEJ-factors, such as DNA ligase IV with SCR7 (NHEJ inhibitor) or the Ku-complex through Ku-specific siRNAs. On the other hand, HDR can be promoted by inhibition of apoptosis or p53-dependent cell-cycle arrest, or the use of cell-cycle-synchronization substances, such as nocodazole (reviewed in Reference [76]). However, only a few of those approaches have been tested with T cells. XL314, a CDC7 cell-cycle kinase inhibitor, has been shown to increase HDR in primary T cells possibly by increasing the retention time in late S or G2 phases of the cell cycle [117]. Treatment of T cells with XL314 post-Cas9 RNP delivery has been shown to increase the total HDR rate 2-fold and 1.2-fold, using ss-DNA and ds-DNA HDR templates, respectively [117]. Fu et al. tested on iPSCs 14 different small molecules with a reported ability to increase HDR-mediated editing [93]. The two most potent NHEJ inhibitors, M3814 and TSA, were then further evaluated on T cells and found to almost completely block NHEJ-mediated DNA repair. A combination of these two compounds promoted AAV-mediated ds-DNA template integration more than 2-fold [93]. These interesting findings require further validation before NHEJ inhibitors find a broader use for CRISPR/Cas-induced and HDR-mediated T-cell modification, especially for the cells used in clinical applications. An alternative could be exposing cells to a cold shock (32 °C for 24 or 48 hours), which was also shown to increase the rate of HDR by 2-to-10-fold in iPSCs and HEK293 cells [170]. Although the mechanism of this effect is not completely understood, it seems that the lower temperature has a thermodynamic effect that stabilizes gene-editing mediators. If proven functional also in T cells, this method would be easily applicable for the production of modified T cells for clinical experiments.

### 5.6. Practical Considerations during Performing HDR-Mediated CRISPR/Cas9 Gene-Editing Experiments

Certain experimental protocol steps seem to be crucial for HDR-mediated CRISPR/Cas9 gene editing. First, it is important to provide the HDR template in the highest amount possible that does not induce toxic effects to the cells. This is especially important when ds-DNA templates are used and available data suggest a narrow range of 1–4 µg per reaction [94,95,114]. Furthermore, it seems that the order by which the ds-DNA is added to the cells can also affect HDR efficiency and cell viability. Roth et al. reported that pre-incubation of RNPs with dsDNA HDR template before adding T cells significantly increases the efficiency of HDR integration at the cost of decreased cell survival. They reported that, for high concentrations of dsDNA HDR templates, T-cell survival is higher when the RNP and cells are mixed before the DNA donor is added immediately before electroporation [94]. If HDR templates are delivered by AAV, viral transduction should be performed immediately after cell electroporation to significantly increase the rate of T-cell transduction [90].

Appropriate timing of transfection or transduction after cell stimulation is an important parameter for successful template delivery into T cells. Several studies have shown that the best time is 2 to 4 days after activation, due to increased cell size and active TCR signaling [25,94,95,115]. The exact optimal time point seems to depend on the activation protocol used in each study and requires experimental validation during the establishment phase.

Finally, although the expression of the integrated genetic information can be determined by using FACS or Western blot, DNA sequencing is of most importance, since some knocked-in genes resulted in protein products with different lengths that deviated from sequences coded by the template [86].

## 6. Conclusions

CRISPR/Cas-mediated gene editing has revolutionized many areas of biology. In T-cell immunology, CRISPR/Cas-mediated modifications have facilitated crucial important insights into T-cell function. More importantly, they have also enabled the production of novel adoptive T-cell products for the treatment of malignant and chronic viral diseases. In recent years, we have witnessed constant optimization of experimental protocols for the delivery of CRISPR/Cas9 components to the T cells. Current protocols using nucleofection of Cas9 RNPS allow for efficacies of close to 100% for gene deletion and up to 60% for HDR template-mediated gene insertion [82,94,99,114,127,128,129]. Such high efficacies allow for the production of genome-edited T cells for adoptive cell transfer into the tumor or immunosuppressed patients, and they are currently being tested in initial clinical trials. Further standardization of the CRISPR/Cas systems holds a promise to solve some of the current remaining challenges, such as excessive cell loss due to cargo delivery or the possibility of unwanted off-target effects.

## Figures and Tables

**Table 1:** Overview of currently ongoing clinical studies with CRISPR-engineered T cells (last time accessed on 21 December 2021).

ClinicalTrials.gov Identifier	Official Study Title	Indication of Treatment	Target of Genome Editing	Study Phase
NCT04037566	“A Safety Study of Autologous T Cells Engineered to Target CD19 and CRISPR Gene Edited to Eliminate Endogenous HPK1 (XYF19 CAR-T Cells) for Relapsed or Refractory Haematopoietic Malignancies”	Relapsed or refractory CD19+ leukemia or lymphoma	XYF19 CAR	Phase 1
NCT03545815	“Phase I Study to Evaluate Treatment of CRISPR-Cas9 Mediated PD-1 and TCR Gene -knocked Out Chimeric Antigen Receptor (CAR) T Cells in Patients with Mesothelin Positive Multiple Solid Tumors”	Solid tumors	KO of PD-1 and TCR	Phase 1
NCT04502446	“A Phase 1, Open-Label, Multicenter, Dose Escalation and Cohort Expansion Study of the Safety and Efficacy of Anti-CD70 Allogeneic CRISPR-Cas9-Engineered T Cells (CTX130) in Subjects with Relapsed or Refractory T or B Cell Malignancies”	Relapsed or refractory T- or B-cell malignancies	CTX130 CAR	Phase 1
NCT04637763	“A Phase 1, Multicenter, Open-Label Study of CB-010, a CRISPR-Edited Allogeneic Anti-CD19 CAR-T Cell Therapy in Patients with Relapsed/Refractory B Cell Non-Hodgkin Lymphoma (ANTLER)”	Relapsed or refractory B-cell non-Hodgkin lymphoma	CB-010 CAR	Phase 1
NCT03398967	“Phase I/II Study to Evaluate Treatment of Relapsed or Refractory Leukemia and Lymphoma with Universal CRISPR-Cas9 Gene-Editing CAR-T Cells Targeting CD19 and CD20 or CD22”	B-cell leukemia; B-cell lymphoma	CAR (CD19 and CD20 or CD22)	Phase 1 and Phase 2
NCT04438083	“A Phase 1 Dose Escalation and Cohort Expansion Study of the Safety and Efficacy of Allogeneic CRISPR-Cas9-Engineered T Cells (CTX130) in Subjects with Advanced, Relapsed or Refractory Renal Cell Carcinoma with Clear Cell Differentiation”	Refractory renal cell carcinoma	CTX13 CAR	Phase 1
NCT04244656	“A Phase 1 Dose Escalation and Cohort Expansion Study of the Safety and Efficacy of Anti-BCMA Allogeneic CRISPR-Cas9-Engineered T Cells (CTX120) in Subjects with Relapsed or Refractory”	Multiple myeloma	CTX120 CAR	Phase 1
NCT04035434	“A Phase 1 Dose Escalation and Cohort Expansion Study of the Safety and Efficacy of Allogeneic CRISPR-Cas9-Engineered T Cells (CTX110) in Subjects with Relapsed or Refractory B-Cell Malignancies (CARBON)”	B-cell malignancy; non-Hodgkin lymphoma; B-cell lymphoma; adult B-cell acute lymphoblastic leukemia	CTX110 CAR	Phase 1
NCT04557436	“Phase 1, Open Label Study of CRISPR-CAR Genome Edited T Cells (PBLTT52CAR19) in Relapsed/Refractory B Cell Acute Lymphoblastic Leukaemia”	B acute lymphoblastic leukemia	PBLTT52CAR19	Phase 1
NCT03166878	“Phase I/II Study to Determine the Safety, Tolerability, Biological Activity and Efficacy of Universal CRISPR-Cas9 Gene-Editing CAR-T Cells Targeting CD19(UCART019) in Patients with Relapsed or Refractory CD19+ Leukemia and Lymphoma”	B-Cell leukemia; B-Cell lymphoma	UCART019 CAR	Phase 1 and Phase 2
NCT04417764	“Safety and Effect Assessment of TACE in Combination with Autologous PD-1 Knockout Engineered T Cells by Percutaneous Infusion in the Patients with Advanced Hepatocellular Carcinoma”	Advanced hepatocellular carcinoma	PD-1 KO	Phase 1
NCT05066165	“Phase 1/2a, Single Dose Study Investigating NTLA-5001 in Subjects with Acute Myeloid Leukemia”	Acute myeloid leukemia	NTLA-5001 CAR	Phase 1 and Phase 2
NCT03044743	“A Phase I/II Trial of PD-1 Knockout EBV-CTLs for Advanced Stage EBV Associated Malignancies”	Carcinoma; T-Cell lymphoma; adult Hodgkin lymphoma; diffuse large B-Cell lymphoma	PD-1 KO in EBV-specific T cells	Phase 1 and Phase 2

## Data Availability

Not applicable.

## References

[1] Linares L., Sanclemente G., Cervera C., Hoyo I., Cofán F., Ricart M.J., Pérez-Villa F., Navasa M., Marcos M.A., Antón A. (2011). Influence of cytomegalovirus disease in outcome of solid organ transplant patients. Transplant. Proc..

[2] Beam E., Razonable R.R. (2012). Cytomegalovirus in solid organ transplantation: Epidemiology, prevention, and treatment. Curr. Infect. Dis. Rep..

[3] Razonable R.R., Humar A. (2013). AST Infectious Diseases Community of Practice Cytomegalovirus in solid organ transplantation. Am. J. Transplant..

[4] Labani-Motlagh A., Ashja-Mahdavi M., Loskog A. (2020). The Tumor Microenvironment: A Milieu Hindering and Obstructing Antitumor Immune Responses. Front. Immunol..

[5] Waldman A.D., Fritz J.M., Lenardo M.J. (2020). A guide to cancer immunotherapy: From T cell basic science to clinical practice. Nat. Rev. Immunol..

[6] He X., Xu C. (2020). Immune checkpoint signaling and cancer immunotherapy. Cell Res..

[7] Mehdizadeh S., Bayatipoor H., Pashangzadeh S., Jafarpour R., Shojaei Z., Motallebnezhad M. (2021). Immune checkpoints and cancer development: Therapeutic implications and future directions. Pathol. Res. Pract..

[8] Perica K., Varela J.C., Oelke M., Schneck J. (2015). Adoptive T cell immunotherapy for cancer. Rambam Maimonides Med. J..

[9] Walter E.A., Greenberg P.D., Gilbert M.J., Finch R.J., Watanabe K.S., Thomas E.D., Riddell S.R. (1995). Reconstitution of cellular immunity against cytomegalovirus in recipients of allogeneic bone marrow by transfer of T-cell clones from the donor. N. Engl. J. Med..

[10] Einsele H., Roosnek E., Rufer N., Sinzger C., Riegler S., Löffler J., Grigoleit U., Moris A., Rammensee H.-G., Kanz L. (2002). Infusion of cytomegalovirus (CMV)-specific T cells for the treatment of CMV infection not responding to antiviral chemotherapy. Blood.

[11] Cobbold M., Khan N., Pourgheysari B., Tauro S., McDonald D., Osman H., Assenmacher M., Billingham L., Steward C., Crawley C. (2005). Adoptive transfer of cytomegalovirus-specific CTL to stem cell transplant patients after selection by HLA-peptide tetramers. J. Exp. Med..

[12] Feuchtinger T., Opherk K., Bethge W.A., Topp M.S., Schuster F.R., Weissinger E.M., Mohty M., Or R., Maschan M., Schumm M. (2010). Adoptive transfer of pp65-specific T cells for the treatment of chemorefractory cytomegalovirus disease or reactivation after haploidentical and matched unrelated stem cell transplantation. Blood.

[13] Peggs K.S., Thomson K., Samuel E., Dyer G., Armoogum J., Chakraverty R., Pang K., Mackinnon S., Lowdell M.W. (2011). Directly selected cytomegalovirus-reactive donor T cells confer rapid and safe systemic reconstitution of virus-specific immunity following stem cell transplantation. Clin. Infect. Dis..

[14] Rosenberg S.A., Packard B.S., Aebersold P.M., Solomon D., Topalian S.L., Toy S.T., Simon P., Lotze M.T., Yang J.C., Seipp C.A. (1988). Use of tumor-infiltrating lymphocytes and interleukin-2 in the immunotherapy of patients with metastatic melanoma. A preliminary report. N. Engl. J. Med..

[15] Rosenberg S.A., Yannelli J.R., Yang J.C., Topalian S.L., Schwartzentruber D.J., Weber J.S., Parkinson D.R., Seipp C.A., Einhorn J.H., White D.E. (1994). Treatment of patients with metastatic melanoma with autologous tumor-infiltrating lymphocytes and interleukin 2. J. Natl. Cancer Inst..

[16] Rooney C.M., Smith C.A., Ng C.Y.C., Loftin S.K., Sixbey J.W., Gan Y., Srivastava D.K., Bowman L.C., Krance R.A., Brenner M.K. (1998). Infusion of cytotoxic T cells for the prevention and treatment of Epstein-Barr virus-induced lymphoma in allogeneic transplant recipients. Blood.

[17] Weiden P.L., Flournoy N., Thomas E.D., Prentice R., Fefer A., Buckner C.D., Storb R. (1979). Antileukemic effect of graft-versus-host disease in human recipients of allogeneic-marrow grafts. N. Engl. J. Med..

[18] Dudley M.E., Yang J.C., Sherry R., Hughes M.S., Royal R., Kammula U., Robbins P.F., Huang J.P., Citrin D.E., Leitman S.F. (2008). Adoptive cell therapy for patients with metastatic melanoma: Evaluation of intensive myeloablative chemoradiation preparative regimens. J. Clin. Oncol..

[19] Rosenberg S.A., Yang J.C., Sherry R.M., Kammula U.S., Hughes M.S., Phan G.Q., Citrin D.E., Restifo N.P., Robbins P.F., Wunderlich J.R. (2011). Durable complete responses in heavily pretreated patients with metastatic melanoma using T-cell transfer immunotherapy. Clin. Cancer Res..

[20] Hong M., Clubb J.D., Chen Y.Y. (2020). Engineering CAR-T Cells for Next-Generation Cancer Therapy. Cancer Cell.

[21] Annesley C.E., Summers C., Ceppi F., Gardner R.A. (2018). The Evolution and Future of CAR T Cells for B-Cell Acute Lymphoblastic Leukemia. Clin. Pharmacol. Ther..

[22] Lulla P.D., Hill L.C., Ramos C.A., Heslop H.E. (2018). The use of chimeric antigen receptor T cells in patients with non-Hodgkin lymphoma. Clin. Adv. Hematol. Oncol..

[23] Hay K.A., Turtle C.J. (2017). Chimeric Antigen Receptor (CAR) T Cells: Lessons Learned from Targeting of CD19 in B-Cell Malignancies. Drugs.

[24] Davila M.L., Sadelain M. (2016). Biology and clinical application of CAR T cells for B cell malignancies. Int. J. Hematol..

[25] Harris E., Elmer J.J. (2021). Optimization of electroporation and other non-viral gene delivery strategies for T cells. Biotechnol. Prog..

[26] Xu C.L., Ruan M.Z.C., Mahajan V.B., Tsang S.H. (2019). Viral Delivery Systems for CRISPR. Viruses.

[27] Cruz P.E., Almeida J.S., Murphy P.N., Moreira J.L., Carrondo M.J.T. (2000). Modeling retrovirus production for gene therapy. 1. Determination Of optimal bioreaction mode and harvest strategy. Biotechnol. Prog..

[28] Levine B.L., Miskin J., Wonnacott K., Keir C. (2017). Global Manufacturing of CAR T Cell Therapy. Mol. Ther. Methods Clin. Dev..

[29] Verhoeyen E., Costa C., Cosset F.-L. (2009). Lentiviral vector gene transfer into human T cells. Methods Mol. Biol..

[30] Gill S., June C.H. (2015). Going viral: Chimeric antigen receptor T-cell therapy for hematological malignancies. Immunol. Rev..

[31] Carroll D. (2017). Focus: Genome Editing: Genome Editing: Past, Present, and Future. Yale J. Biol. Med..

[32] Kim Y.G., Cha J., Chandrasegaran S. (1996). Hybrid restriction enzymes: Zinc finger fusions to Fok I cleavage domain. Proc. Natl. Acad. Sci. USA.

[33] Bibikova M., Carroll D., Segal D.J., Trautman J.K., Smith J., Kim Y.G., Chandrasegaran S. (2001). Stimulation of homologous recombination through targeted cleavage by chimeric nucleases. Mol. Cell. Biol..

[34] Miller J., McLachlan A.D., Klug A. (1985). Repetitive zinc-binding domains in the protein transcription factor IIIA from Xenopus oocytes. EMBO J..

[35] Pabo C.O., Peisach E., Grant R.A. (2001). Design and selection of novel Cys2His2 zinc finger proteins. Annu. Rev. Biochem..

[36] Urnov F.D., Rebar E.J., Holmes M.C., Zhang H.S., Gregory P.D. (2010). Genome editing with engineered zinc finger nucleases. Nat. Rev. Genet..

[37] Boch J., Scholze H., Schornack S., Landgraf A., Hahn S., Kay S., Lahaye T., Nickstadt A., Bonas U. (2009). Breaking the code of DNA binding specificity of TAL-type III effectors. Science.

[38] Miller J.C., Tan S., Qiao G., Barlow K.A., Wang J., Xia D.F., Meng X., Paschon D.E., Leung E., Hinkley S.J. (2011). A TALE nuclease architecture for efficient genome editing. Nat. Biotechnol..

[39] Bedell V.M., Wang Y., Campbell J.M., Poshusta T.L., Starker C.G., Krug R.G., Tan W., Penheiter S.G., Ma A.C., Leung A.Y.H. (2012). In vivo genome editing using a high-efficiency TALEN system. Nature.

[40] Mussolino C., Morbitzer R., Lütge F., Dannemann N., Lahaye T., Cathomen T. (2011). A novel TALE nuclease scaffold enables high genome editing activity in combination with low toxicity. Nucleic Acids Res..

[41] Gupta R.M., Musunuru K. (2014). Expanding the genetic editing tool kit: ZFNs, TALENs, and CRISPR-Cas9. J. Clin. Investig..

[42] Provasi E., Genovese P., Lombardo A., Magnani Z., Liu P.-Q., Reik A., Chu V., Paschon D.E., Zhang L., Kuball J. (2012). Editing T cell specificity towards leukemia by zinc finger nucleases and lentiviral gene transfer. Nat. Med..

[43] Alzubi J., Lock D., Rhiel M., Schmitz S., Wild S., Mussolino C., Hildenbeutel M., Brandes C., Rositzka J., Lennartz S. (2021). Automated generation of gene-edited CAR T cells at clinical scale. Mol. Ther. Methods Clin. Dev..

[44] Li H., Yang Y., Hong W., Huang M., Wu M., Zhao X. (2020). Applications of genome editing technology in the targeted therapy of human diseases: Mechanisms, advances and prospects. Signal Transduct. Target. Ther..

[45] Jansen R., van Embden J.D.A., Gaastra W., Schouls L.M. (2002). Identification of genes that are associated with DNA repeats in prokaryotes. Mol. Microbiol..

[46] Sorek R., Kunin V., Hugenholtz P. (2008). CRISPR—A widespread system that provides acquired resistance against phages in bacteria and archaea. Nat. Rev. Microbiol..

[47] Deveau H., Garneau J.E., Moineau S. (2010). CRISPR/Cas system and its role in phage-bacteria interactions. Annu. Rev. Microbiol..

[48] Van der Oost J., Jore M.M., Westra E.R., Lundgren M., Brouns S.J.J. (2009). CRISPR-based adaptive and heritable immunity in prokaryotes. Trends Biochem. Sci..

[49] Cong L., Ran F.A., Cox D., Lin S., Barretto R., Habib N., Hsu P.D., Wu X., Jiang W., Marraffini L.A. (2013). Multiplex Genome Engineering Using CRISPR/Cas Systems. Science.

[50] Jinek M., Chylinski K., Fonfara I., Hauer M., Doudna J.A., Charpentier E. (2012). A programmable dual-RNA-guided DNA endonuclease in adaptive bacterial immunity. Science.

[51] Mali P., Yang L., Esvelt K.M., Aach J., Guell M., DiCarlo J.E., Norville J.E., Church G.M. (2013). RNA-guided human genome engineering via Cas9. Science.

[52] Makarova K.S., Wolf Y.I., Iranzo J., Shmakov S.A., Alkhnbashi O.S., Brouns S.J.J., Charpentier E., Cheng D., Haft D.H., Horvath P. (2020). Evolutionary classification of CRISPR-Cas systems: A burst of class 2 and derived variants. Nat. Rev. Microbiol..

[53] Nozawa T., Furukawa N., Aikawa C., Watanabe T., Haobam B., Kurokawa K., Maruyama F., Nakagawa I. (2011). CRISPR inhibition of prophage acquisition in Streptococcus pyogenes. PLoS ONE.

[54] Gasiunas G., Siksnys V. (2013). RNA-dependent DNA endonuclease Cas9 of the CRISPR system: Holy Grail of genome editing?. Trends Microbiol..

[55] Hsu P.D., Scott D.A., Weinstein J.A., Ran F.A., Konermann S., Agarwala V., Li Y., Fine E.J., Wu X., Shalem O. (2013). DNA targeting specificity of RNA-guided Cas9 nucleases. Nat. Biotechnol..

[56] Scully R., Panday A., Elango R., Willis N.A. (2019). DNA double-strand break repair-pathway choice in somatic mammalian cells. Nat. Rev. Mol. Cell Biol..

[57] Davis A.J., Chen D.J. (2013). DNA double strand break repair via non-homologous end-joining. Transl. Cancer Res..

[58] Zhao B., Rothenberg E., Ramsden D.A., Lieber M.R. (2020). The molecular basis and disease relevance of non-homologous DNA end joining. Nat. Rev. Mol. Cell Biol..

[59] Liang F., Han M., Romanienko P.J., Jasin M. (1998). Homology-directed repair is a major double-strand break repair pathway in mammalian cells. Proc. Natl. Acad. Sci. USA.

[60] Lieber M.R. (2008). The mechanism of human nonhomologous DNA end joining. J. Biol. Chem..

[61] Li Z., Zhang W., Chen Y., Guo W., Zhang J., Tang H., Xu Z., Zhang H., Tao Y., Wang F. (2016). Impaired DNA double-strand break repair contributes to the age-associated rise of genomic instability in humans. Cell Death Differ..

[62] Guo T., Feng Y.L., Xiao J.J., Liu Q., Sun X.N., Xiang J.F., Kong N., Liu S.C., Chen G.Q., Wang Y. (2018). Harnessing accurate non-homologous end joining for efficient precise deletion in CRISPR/Cas9-mediated genome editing. Genome Biol..

[63] Wang H., Xu X. (2017). Microhomology-mediated end joining: New players join the team. Cell Biosci..

[64] Maréchal A., Zou L. (2013). DNA damage sensing by the ATM and ATR kinases. Cold Spring Harb. Perspect. Biol..

[65] Rogakou E.P., Pilch D.R., Orr A.H., Ivanova V.S., Bonner W.M. (1998). DNA double-stranded breaks induce histone H2AX phosphorylation on serine 139. J. Biol. Chem..

[66] Rogakou E.P., Boon C., Redon C., Bonner W.M. (1999). Megabase chromatin domains involved in DNA double-strand breaks in vivo. J. Cell Biol..

[67] Burma S., Chen B.P., Murphy M., Kurimasa A., Chen D.J. (2001). ATM phosphorylates histone H2AX in response to DNA double-strand breaks. J. Biol. Chem..

[68] Stope M.B. (2021). Phosphorylation of histone H2A.X as a DNA-associated biomarker (Review). World Acad. Sci. J..

[69] Marini F., Rawal C.C., Liberi G., Pellicioli A. (2019). Regulation of DNA Double Strand Breaks Processing: Focus on Barriers. Front. Mol. Biosci..

[70] Renkawitz J., Lademann C.A., Jentsch S. (2014). Mechanisms and principles of homology search during recombination. Nat. Rev. Mol. Cell Biol..

[71] Bhat K.P., Cortez D. (2018). RPA and RAD51: Fork reversal, fork protection, and genome stability. Nat. Struct. Mol. Biol..

[72] Pastwa E., Błasiak J. (2003). Non-homologous DNA end joining. Acta Biochim. Pol..

[73] Mao Z., Bozzella M., Seluanov A., Gorbunova V. (2008). Comparison of nonhomologous end joining and homologous recombination in human cells. DNA Repair.

[74] Zhang J.P., Li X.L., Li G.H., Chen W., Arakaki C., Botimer G.D., Baylink D., Zhang L., Wen W., Fu Y.W. (2017). Efficient precise knockin with a double cut HDR donor after CRISPR/Cas9-mediated double-stranded DNA cleavage. Genome Biol..

[75] Carballar R., Martínez-Láinez J.M., Samper B., Bru S., Bállega E., Mirallas O., Ricco N., Clotet J., Jiménez J. (2020). CDK-mediated Yku80 Phosphorylation Regulates the Balance Between Non-homologous End Joining (NHEJ) and Homologous Directed Recombination (HDR). J. Mol. Biol..

[76] Yang H., Ren S., Yu S., Pan H., Li T., Ge S., Zhang J., Xia N. (2020). Methods Favoring Homology-Directed Repair Choice in Response to CRISPR/Cas9 Induced-Double Strand Breaks. Int. J. Mol. Sci..

[77] Buis J., Stoneham T., Spehalski E., Ferguson D.O. (2012). Mre11 regulates CtIP-dependent double-strand break repair by interaction with CDK2. Nat. Struct. Mol. Biol..

[78] Huertas P., Jackason S.P. (2009). Human CtIP mediates cell cycle control of DNA end resection and double strand break repair. J. Biol. Chem..

[79] Ismail I.H., Gagné J.-P., Genois M.-M., Strickfaden H., McDonald D., Xu Z., Poirier G.G., Masson J.-Y., Hendzel M.J. (2015). The RNF138 E3 ligase displaces Ku to promote DNA end resection and regulate DNA repair pathway choice. Nat. Cell Biol..

[80] Kakarougkas A., Jeggo P.A. (2014). DNA DSB repair pathway choice: An orchestrated handover mechanism. Br. J. Radiol..

[81] Rathmell J.C., Farkash E.A., Gao W., Thompson C.B. (2001). IL-7 Enhances the Survival and Maintains the Size of Naive T Cells. J. Immunol..

[82] Seki A., Rutz S. (2018). Optimized RNP transfection for highly efficient CRISPR/Cas9-mediated gene knockout in primary T cells. J. Exp. Med..

[83] Nüssing S., House I.G., Kearney C.J., Chen A.X.Y., Vervoort S.J., Beavis P.A., Oliaro J., Johnstone R.W., Trapani J.A., Parish I.A. (2020). Efficient CRISPR/Cas9 Gene Editing in Uncultured Naive Mouse T Cells for In Vivo Studies. J. Immunol..

[84] Majumder S., Jugovic I., Saul D., Bell L., Hundhausen N., Seal R., Beilhack A., Rosenwald A., Mougiakakos D., Berberich-Siebelt F. (2021). Rapid and Efficient Gene Editing for Direct Transplantation of Naive Murine Cas9^+^ T Cells. Front. Immunol..

[85] Verkuijl S.A., Rots M.G. (2019). The influence of eukaryotic chromatin state on CRISPR–Cas9 editing efficiencies. Curr. Opin. Biotechnol..

[86] Borowicz P., Chan H., Medina D., Gumpelmair S., Kjelstrup H., Spurkland A. (2020). A simple and efficient workflow for generation of knock-in mutations in Jurkat T cells using CRISPR/Cas9. Scand. J. Immunol..

[87] Liu M., Rehman S., Tang X., Gu K., Fan Q., Chen D., Ma W. (2019). Methodologies for improving HDR efficiency. Front. Genet..

[88] Ghaffari S., Torabi-Rahvar M., Omidkhoda A., Ahmadbeigi N. (2019). Impact of various culture conditions on ex vivo expansion of polyclonal T cells for adoptive immunotherapy. APMIS.

[89] Bere A., Denny L., Hanekom W., Burgers W.A., Passmore J.A.S. (2010). Comparison of polyclonal expansion methods to improve the recovery of cervical cytobrush-derived T cells from the female genital tract of HIV-infected women. J. Immunol. Methods.

[90] Lam A.J., Lin D.T.S., Gillies J.K., Uday P., Pesenacker A.M., Kobor M.S., Levings M.K. (2021). Optimized CRISPR-mediated gene knockin reveals FOXP3-independent maintenance of human Treg identity. Cell Rep..

[91] Xu H., Wang N., Cao W., Huang L., Zhou J., Sheng L. (2018). Influence of various medium environment to in vitro human T cell culture. Vitr. Cell. Dev. Biol.-Anim..

[92] Hendel A., Bak R.O., Clark J.T., Kennedy A.B., Ryan D.E., Roy S., Steinfeld I., Lunstad B.D., Kaiser R.J., Wilkens A.B. (2015). Chemically modified guide RNAs enhance CRISPR-Cas genome editing in human primary cells. Nat. Biotechnol..

[93] Fu Y.W., Dai X.Y., Wang W.T., Yang Z.X., Zhao J.J., Zhang J.P., Wen W., Zhang F., Oberg K.C., Zhang L. (2021). Dynamics and competition of CRISPR-Cas9 ribonucleoproteins and AAV donor-mediated NHEJ, MMEJ and HDR editing. Nucleic Acids Res..

[94] Roth T.L., Puig-Saus C., Yu R., Shifrut E., Carnevale J., Li P.J., Hiatt J., Saco J., Krystofinski P., Li H. (2018). Reprogramming human T cell function and specificity with non-viral genome targeting. Nature.

[95] Kath J., Du W., Thommandru B., Turk R., Amini L., Stein M., Zittel T., Martini S., Ostendorf L., Wilhelm A. (2021). Fast, Efficient and Virus-Free Generation of TRAC-Replaced CAR T Cells. SSRN Electron. J..

[96] Ghaffari S., Torabi-Rahvar M., Aghayan S., Jabbarpour Z., Moradzadeh K., Omidkhoda A., Ahmadbeigi N. (2021). Optimizing interleukin-2 concentration, seeding density and bead-to-cell ratio of T-cell expansion for adoptive immunotherapy. BMC Immunol..

[97] Raulf M. (2019). T cell: Primary culture from peripheral blood. Methods in Molecular Biology.

[98] Wang W., Ai X. (2021). Primary culture of immature, naïve mouse CD4^+^ T cells. STAR Protoc..

[99] Martens R., Permanyer M., Werth K., Yu K., Braun A., Halle O., Halle S., Patzer G.E., Bošnjak B., Kiefer F. (2020). Efficient homing of T cells via afferent lymphatics requires mechanical arrest and integrin-supported chemokine guidance. Nat. Commun..

[100] Liu G., Zhang Y., Zhang T. (2020). Computational approaches for effective CRISPR guide RNA design and evaluation. Comput. Struct. Biotechnol. J..

[101] Cui Y., Xu J., Cheng M., Liao X., Peng S. (2018). Review of CRISPR/Cas9 sgRNA Design Tools. Interdiscip. Sci..

[102] Huang M.C., Cheong W.C., Lim L.S., Li M.-H. (2012). A simple, high sensitivity mutation screening using Ampligase mediated T7 endonuclease I and Surveyor nuclease with microfluidic capillary electrophoresis. Electrophoresis.

[103] Cho S.W., Kim S., Kim Y., Kweon J., Kim H.S., Bae S., Kim J.-S. (2014). Analysis of off-target effects of CRISPR/Cas-derived RNA-guided endonucleases and nickases. Genome Res..

[104] Tsai S.Q., Zheng Z., Nguyen N.T., Liebers M., Topkar V.V., Thapar V., Wyvekens N., Khayter C., Iafrate A.J., Le L.P. (2015). GUIDE-seq enables genome-wide profiling of off-target cleavage by CRISPR-Cas nucleases. Nat. Biotechnol..

[105] Zhang X.-H., Tee L.Y., Wang X.-G., Huang Q.-S., Yang S.-H. (2015). Off-target Effects in CRISPR/Cas9-mediated Genome Engineering. Mol. Ther. Nucleic Acids.

[106] Vakulskas C.A., Behlke M.A. (2019). Evaluation and Reduction of CRISPR Off-Target Cleavage Events. Nucleic Acid Ther..

[107] DeBruin K.A., Krassowska W. (1999). Modeling electroporation in a single cell. I. Effects Of field strength and rest potential. Biophys. J..

[108] Luecke S., Holleufer A., Christensen M.H., Jønsson K.L., Boni G.A., Sørensen L.K., Johannsen M., Jakobsen M.R., Hartmann R., Paludan S.R. (2017). cGAS is activated by DNA in a length-dependent manner. EMBO Rep..

[109] Zierhut C., Yamaguchi N., Paredes M., Luo J.-D., Carroll T., Funabiki H. (2019). The Cytoplasmic DNA Sensor cGAS Promotes Mitotic Cell Death. Cell.

[110] Kim S., Kim D., Cho S.W., Kim J., Kim J.-S. (2014). Highly efficient RNA-guided genome editing in human cells via delivery of purified Cas9 ribonucleoproteins. Genome Res..

[111] Shen B., Zhang W., Zhang J., Zhou J., Wang J., Chen L., Wang L., Hodgkins A., Iyer V., Huang X. (2014). Efficient genome modification by CRISPR-Cas9 nickase with minimal off-target effects. Nat. Methods.

[112] Miller J.B., Zhang S., Kos P., Xiong H., Zhou K., Perelman S.S., Zhu H., Siegwart D.J. (2017). Non-Viral CRISPR/Cas Gene Editing In Vitro and In Vivo Enabled by Synthetic Nanoparticle Co-Delivery of Cas9 mRNA and sgRNA. Angew. Chem. Int. Ed. Engl..

[113] Michieletto D., Lusic M., Marenduzzo D., Orlandini E. (2019). Physical principles of retroviral integration in the human genome. Nat. Commun..

[114] Schumann K., Lin S., Boyer E., Simeonov D.R., Subramaniam M., Gate R.E., Haliburton G.E., Ye C.J., Bluestone J.A., Doudna J.A. (2015). Generation of knock-in primary human T cells using Cas9 ribonucleoproteins. Proc. Natl. Acad. Sci. USA.

[115] Nguyen D.N., Roth T.L., Li P.J., Chen P.A., Apathy R., Mamedov M.R., Vo L.T., Tobin V.R., Goodman D., Shifrut E. (2020). Polymer-stabilized Cas9 nanoparticles and modified repair templates increase genome editing efficiency. Nat. Biotechnol..

[116] Schubert M.S., Thommandru B., Woodley J., Turk R., Yan S., Kurgan G., McNeill M.S., Rettig G.R. (2021). Optimized design parameters for CRISPR Cas9 and Cas12a homology-directed repair. Sci. Rep..

[117] Kotowski M., Sharma S. (2020). CRISPR-Based Editing Techniques for Genetic Manipulation of Primary T Cells. Methods Protoc..

[118] Atsavapranee E.S., Billingsley M.M., Mitchell M.J. (2021). Delivery technologies for T cell gene editing: Applications in cancer immunotherapy. EBioMedicine.

[119] Yip B.H. (2020). Recent Advances in CRISPR/Cas9 Delivery Strategies. Biomolecules.

[120] Horii T., Arai Y., Yamazaki M., Morita S., Kimura M., Itoh M., Abe Y., Hatada I. (2014). Validation of microinjection methods for generating knockout mice by CRISPR/Cas-mediated genome engineering. Sci. Rep..

[121] Raveux A., Vandormael-Pournin S., Cohen-Tannoudji M. (2017). Optimization of the production of knock-in alleles by CRISPR/Cas9 microinjection into the mouse zygote. Sci. Rep..

[122] Rahimmanesh I., Totonchi M., Khanahmad H. (2020). The challenging nature of primary T lymphocytes for transfection: Effect of protamine sulfate on the transfection efficiency of chemical transfection reagents. Res. Pharm. Sci..

[123] Bošnjak B., Permanyer M., Sethi M.K., Galla M., Maetzig T., Heinemann D., Willenzon S., Förster R., Heisterkamp A., Kalies S. (2018). CRISPR/Cas9 Genome Editing Using Gold-Nanoparticle-Mediated Laserporation. Adv. Biosyst..

[124] Boukany P.E., Morss A., Liao W.-C., Henslee B., Jung H., Zhang X., Yu B., Wang X., Wu Y., Li L. (2011). Nanochannel electroporation delivers precise amounts of biomolecules into living cells. Nat. Nanotechnol..

[125] Kumar P., Nagarajan A., Uchil P.D. (2019). Electroporation. Cold Spring Harb. Protoc..

[126] Eyquem J., Mansilla-Soto J., Giavridis T., van der Stegen S.J.C., Hamieh M., Cunanan K.M., Odak A., Gönen M., Sadelain M. (2017). Targeting a CAR to the TRAC locus with CRISPR/Cas9 enhances tumour rejection. Nature.

[127] Schober K., Müller T.R., Gökmen F., Grassmann S., Effenberger M., Poltorak M., Stemberger C., Schumann K., Roth T.L., Marson A. (2019). Orthotopic replacement of T-cell receptor α- and β-chains with preservation of near-physiological T-cell function. Nat. Biomed. Eng..

[128] Paul B., Ibarra G.S.R., Hubbard N., Einhaus T., Astrakhan A., Rawlings D.J., Kiem H.-P., Peterson C.W. (2018). Efficient Enrichment of Gene-Modified Primary T Cells via CCR5-Targeted Integration of Mutant Dihydrofolate Reductase. Mol. Ther. Methods Clin. Dev..

[129] Roth T.L., Li P.J., Blaeschke F., Nies J.F., Apathy R., Mowery C., Yu R., Nguyen M.L.T., Lee Y., Truong A. (2020). Pooled Knockin Targeting for Genome Engineering of Cellular Immunotherapies. Cell.

[130] Durán V., Grabski E., Hozsa C., Becker J., Yasar H., Monteiro J.T., Costa B., Koller N., Lueder Y., Wiegmann B. (2021). Fucosylated lipid nanocarriers loaded with antibiotics efficiently inhibit mycobacterial propagation in human myeloid cells. J. Control. Release.

[131] Chicaybam L., Sodre A.L., Curzio B.A., Bonamino M.H. (2013). An Efficient Low Cost Method for Gene Transfer to T Lymphocytes. PLoS ONE.

[132] Zhang M., Ma Z., Selliah N., Weiss G., Genin A., Finkel T.H., Cron R.Q. (2014). The impact of Nucleofection^®^ on the activation state of primary human CD4 T cells. J. Immunol. Methods.

[133] DiTommaso T., Cole J.M., Cassereau L., Buggé J.A., Hanson J.L.S., Bridgen D.T., Stokes B.D., Loughhead S.M., Beutel B.A., Gilbert J.B. (2018). Cell engineering with microfluidic squeezing preserves functionality of primary immune cells in vivo. Proc. Natl. Acad. Sci. USA.

[134] D’Astolfo D.S., Pagliero R.J., Pras A., Karthaus W.R., Clevers H., Prasad V., Lebbink R.J., Rehmann H., Geijsen N. (2015). Efficient intracellular delivery of native proteins. Cell.

[135] Kholosy W.M., Visscher M., Ogink K., Buttstedt H., Griffin K., Beier A., Gerlach J.P., Molenaar J.J., Geijsen N., de Boer M. (2021). Simple, fast and efficient iTOP-mediated delivery of CRISPR/Cas9 RNP in difficult-to-transduce human cells including primary T cells. J. Biotechnol..

[136] Agrotis A., Ketteler R. (2015). A new age in functional genomics using CRISPR/Cas9 in arrayed library screening. Front. Genet..

[137] Shang W., Jiang Y., Boettcher M., Ding K., Mollenauer M., Liu Z., Wen X., Liu C., Hao P., Zhao S. (2018). Genome-wide CRISPR screen identifies FAM49B as a key regulator of actin dynamics and T cell activation. Proc. Natl. Acad. Sci. USA.

[138] Cortez J.T., Montauti E., Shifrut E., Gatchalian J., Zhang Y., Shaked O., Xu Y., Roth T.L., Simeonov D.R., Zhang Y. (2020). CRISPR screen in regulatory T cells reveals modulators of Foxp3. Nature.

[139] Chen Z., Arai E., Khan O., Zhang Z., Ngiow S.F., He Y., Huang H., Manne S., Cao Z., Baxter A.E. (2021). In vivo CD8^+^ T cell CRISPR screening reveals control by Fli1 in infection and cancer. Cell.

[140] Yi M., Niu M., Xu L., Luo S., Wu K. (2021). Regulation of PD-L1 expression in the tumor microenvironment. J. Hematol. Oncol..

[141] Konishi J., Yamazaki K., Azuma M., Kinoshita I., Dosaka-Akita H., Nishimura M. (2004). B7-H1 Expression on Non-Small Cell Lung Cancer Cells and Its Relationship with Tumor-Infiltrating Lymphocytes and Their PD-1 Expression. Clin. Cancer Res..

[142] Hamanishi J., Mandai M., Iwasaki M., Okazaki T., Tanaka Y., Yamaguchi K., Higuchi T., Yagi H., Takakura K., Minato N. (2007). Programmed cell death 1 ligand 1 and tumor-infiltrating CD8^+^ T lymphocytes are prognostic factors of human ovarian cancer. Proc. Natl. Acad. Sci. USA.

[143] Zhao Z., Shi L., Zhang W., Han J., Zhang S., Fu Z., Cai J. (2018). CRISPR knock out of programmed cell death protein 1 enhances anti-tumor activity of cytotoxic T lymphocytes. Oncotarget.

[144] Su S., Zou Z., Chen F., Ding N., Du J., Shao J., Li L., Fu Y., Hu B., Yang Y. (2017). CRISPR-Cas9-mediated disruption of PD-1 on human T cells for adoptive cellular therapies of EBV positive gastric cancer. Oncoimmunology.

[145] Choi B.D., Yu X., Castano A.P., Darr H., Henderson D.B., Bouffard A.A., Larson R.C., Scarfò I., Bailey S.R., Gerhard G.M. (2019). CRISPR-Cas9 disruption of PD-1 enhances activity of universal EGFRvIII CAR T cells in a preclinical model of human glioblastoma. J. Immunother. Cancer.

[146] Lu Y., Xue J., Deng T., Zhou X., Yu K., Deng L., Huang M., Yi X., Liang M., Wang Y. (2020). Safety and feasibility of CRISPR-edited T cells in patients with refractory non-small-cell lung cancer. Nat. Med..

[147] Stadtmauer E.A., Fraietta J.A., Davis M.M., Cohen A.D., Weber K.L., Lancaster E., Mangan P.A., Kulikovskaya I., Gupta M., Chen F. (2020). CRISPR-engineered T cells in patients with refractory cancer. Science.

[148] Iwai Y., Terawaki S., Ikegawa M., Okazaki T., Honjo T. (2003). PD-1 inhibits antiviral immunity at the effector phase in the liver. J. Exp. Med..

[149] Amini L., Wagner D.L., Rössler U., Zarrinrad G., Wagner L.F., Vollmer T., Wendering D.J., Kornak U., Volk H.D., Reinke P. (2021). CRISPR-Cas9-Edited Tacrolimus-Resistant Antiviral T Cells for Advanced Adoptive Immunotherapy in Transplant Recipients. Mol. Ther..

[150] Jung I.-Y., Kim Y.-Y., Yu H.-S., Lee M., Kim S., Lee J. (2018). CRISPR/Cas9-Mediated Knockout of DGK Improves Antitumor Activities of Human T Cells. Cancer Res..

[151] Giuffrida L., Sek K., Henderson M.A., Lai J., Chen A.X.Y., Meyran D., Todd K.L., Petley E.V., Mardiana S., Mølck C. (2021). CRISPR/Cas9 mediated deletion of the adenosine A2A receptor enhances CAR T cell efficacy. Nat. Commun..

[152] Kamali E., Rahbarizadeh F., Hojati Z., Frödin M. (2021). CRISPR/Cas9-mediated knockout of clinically relevant alloantigenes in human primary T cells. BMC Biotechnol..

[153] Shen M.W., Arbab M., Hsu J.Y., Worstell D., Culbertson S.J., Krabbe O., Cassa C.A., Liu D.R., Gifford D.K., Sherwood R.I. (2018). Predictable and precise template-free CRISPR editing of pathogenic variants. Nature.

[154] Van Overbeek M., Capurso D., Carter M.M., Thompson M.S., Frias E., Russ C., Reece-Hoyes J.S., Nye C., Gradia S., Vidal B. (2016). DNA Repair Profiling Reveals Nonrandom Outcomes at Cas9-Mediated Breaks. Mol. Cell.

[155] Mollanoori H., Teimourian S. (2018). Therapeutic applications of CRISPR/Cas9 system in gene therapy. Biotechnol. Lett..

[156] Firth A.L., Menon T., Parker G.S., Qualls S.J., Lewis B.M., Ke E., Dargitz C.T., Wright R., Khanna A., Gage F.H. (2015). Functional Gene Correction for Cystic Fibrosis in Lung Epithelial Cells Generated from Patient iPSCs. Cell Rep..

[157] Liu Y., Yang Y., Kang X., Lin B., Yu Q., Song B., Gao G., Chen Y., Sun X., Li X. (2017). One-Step Biallelic and Scarless Correction of a β-Thalassemia Mutation in Patient-Specific iPSCs without Drug Selection. Mol. Ther. Nucleic Acids.

[158] Park C.-Y., Kim D.H., Son J.S., Sung J.J., Lee J., Bae S., Kim J.-H., Kim D.-W., Kim J.-S. (2015). Functional Correction of Large Factor VIII Gene Chromosomal Inversions in Hemophilia A Patient-Derived iPSCs Using CRISPR-Cas9. Cell Stem Cell.

[159] Jafari H., Hesami S., Safi M., Ghasemi F., Banan M. (2019). Expression and hydroxyurea-triggered induction of EGFP upon CRISPR/Cas9-mediated integration into the γ-globin gene of K562 cells. Biotechnol. Lett..

[160] Wiebking V., Lee C.M., Mostrel N., Lahiri P., Bak R., Bao G., Roncarolo M.G., Bertaina A., Porteus M.H. (2021). Genome editing of donor-derived T-cells to generate allogenic chimeric antigen receptor-modified T cells: Optimizing αβ T cell-depleted haploidentical hematopoietic stem cell transplantation. Haematologica.

[161] Tatiossian K.J., Clark R.D.E., Huang C., Thornton M.E., Grubbs B.H., Cannon P.M. (2021). Rational Selection of CRISPR-Cas9 Guide RNAs for Homology-Directed Genome Editing. Mol. Ther..

[162] Liang X., Potter J., Kumar S., Ravinder N., Chesnut J.D. (2017). Enhanced CRISPR/Cas9-mediated precise genome editing by improved design and delivery of gRNA, Cas9 nuclease, and donor DNA. J. Biotechnol..

[163] Paquet D., Kwart D., Chen A., Sproul A., Jacob S., Teo S., Olsen K.M., Gregg A., Noggle S., Tessier-Lavigne M. (2016). Efficient introduction of specific homozygous and heterozygous mutations using CRISPR/Cas9. Nature.

[164] O’Brien A.R., Wilson L.O.W., Burgio G., Bauer D.C. (2019). Unlocking HDR-mediated nucleotide editing by identifying high-efficiency target sites using machine learning. Sci. Rep..

[165] Richardson C.D., Ray G.J., DeWitt M.A., Curie G.L., Corn J.E. (2016). Enhancing homology-directed genome editing by catalytically active and inactive CRISPR-Cas9 using asymmetric donor DNA. Nat. Biotechnol..

[166] Okamoto S., Amaishi Y., Maki I., Enoki T., Mineno J. (2019). Highly efficient genome editing for single-base substitutions using optimized ssODNs with Cas9-RNPs. Sci. Rep..

[167] Wang Y., Liu K.I., Sutrisnoh N.A.B., Srinivasan H., Zhang J., Li J., Zhang F., Lalith C.R.J., Xing H., Shanmugam R. (2018). Systematic evaluation of CRISPR-Cas systems reveals design principles for genome editing in human cells. Genome Biol..

[168] Wen W., Quan Z.J., Li S.A., Yang Z.X., Fu Y.W., Zhang F., Li G.H., Zhao M., Yin M.-D., Xu J. (2021). Effective control of large deletions after double-strand breaks by homology-directed repair and dsODN insertion. Genome Biol..

[169] Paix A., Folkmann A., Goldman D.H., Kulaga H., Grzelak M.J., Rasoloson D., Paidemarry S., Green R., Reed R.R., Seydoux G. (2017). Precision genome editing using synthesis-dependent repair of Cas9-induced DNA breaks. Proc. Natl. Acad. Sci. USA.

[170] Guo Q., Mintier G., Ma-Edmonds M., Storton D., Wang X., Xiao X., Kienzle B., Zhao D., Feder J.N. (2018). ‘Cold shock’ increases the frequency of homology directed repair gene editing in induced pluripotent stem cells. Sci. Rep..

